# The frequency and prognostic effect of *TERT* promoter mutation in diffuse gliomas

**DOI:** 10.1186/s40478-017-0465-1

**Published:** 2017-08-29

**Authors:** Yujin Lee, Jaemoon Koh, Seong-Ik Kim, Jae Kyung Won, Chul-Kee Park, Seung Hong Choi, Sung-Hye Park

**Affiliations:** 1 Department of Pathology, Seoul National University Hospital, 103 Daehak-ro, Jongno-gu, Seoul, 110-799 Republic of Korea; 2 Department of Pathology, Seoul National University College of Medicine, Seoul, Republic of Korea; 3 Department of Neurosurgery, Seoul National University, College of Medicine, Seoul, Republic of Korea; 4 Department of Radiology, Seoul National University, College of Medicine, Seoul, Republic of Korea; 5 Neuroscience Institute Seoul National University, College of Medicine, Seoul, Republic of Korea

**Keywords:** Telomerase reverse transcriptase, Alpha-thalassemia/mental retardation syndrome X-linked (ATRX), Anaplastic astrocytoma, Glioblastoma, Isocitrate dehydrogenase (IDH), Oligodendroglioma

## Abstract

Mutations in the telomerase reverse transcriptase gene promoter *(TERTp)* are common in glioblastomas (GBMs) and oligodendrogliomas (ODGs), and therefore, have a key role in tumorigenesis and may be of prognostic value. However, the extent of their prognostic importance in various gliomas is controversial. We studied 168 patients separated into five groups: Group 1: 65 patients with ODG carrying an *IDH1* or *IDH2* mutation (*IDH*-mutant) and 1p/19q–codeletion, Group 2: 23 patients with anaplastic astrocytoma (AA), *IDH*-mutant, Group 3: 13 patients with GBM, *IDH*-mutant, Group 4: 15 patients with AA, *IDH*-wildtype (WT), and Group 5: 52 patients with GBM, *IDH*-WT. *TERTp* mutations were found in 96.9%, 4.4%, 76.9%, 20.0%, and 84.6% of patients in Groups 1, 2, 3, 4, and 5, respectively. The R132H mutation in *IDH1* was found in 60.5% (23/38) of patients in the AA cohort (Groups 2 and 4) and 20.0% (13/65) of patients from our GBM cohort (Groups 3 and 5), whereas all patients with ODG (Group 1) had a mutation either in *IDH1* (*n* = 62) or *IDH2* (*n* = 3). Using Kaplan Meier survival analysis, we found that the *TERTp* mutation was correlated with poor overall survival (OS) in Groups 2 and 4 combined (*P* = 0.001) and in Group 4 (*P* = 0.113), and in multivariate analysis, the *TERTp* mutant group was associated with significantly poor survival in Group 5 (*P* = 0.045). However, *IDH* mutation, *MGMT* methylation, and younger patient age (<55 years old) were significantly correlated with favorable OS (all *P* < 0.05) in our cohort of astrocytic and ODGs. In patients with ODG (Group 1), mutant *IDH* and *TERTp* did not have prognostic value because these mutations were universally present. Based on the revised 2016 WHO classification of gliomas, we found that *TERTp* mutation was frequently present in patients with GBM or ODG and because it was strongly correlated with poor survival outcome in patients with *IDH*-WT GBM in multivariate analysis, it may be of prognostic value in this subgroup of patients with gliomas.

## Introduction

Gliomas are the most common primary malignant tumor of the central nervous system (CNS), and are comprised of diffuse astrocytic and oligodendroglial tumors (ODG). Significant research in the pathogenesis of gliomas revealed that various morphological phenotypes are related to underlying molecular genetic variations [4, 26]. This understanding has resulted in the restructuring of glioma classification with the incorporation of genetically defined entities and histology [19].

Of the many molecular changes found in gliomas, mutations in *IDH1* or *IDH2* occur early during glioma formation, and are associated with better overall survival (OS). In addition, glioma subtypes can be identified by the *IDH1/IDH2* mutation [11, 27]. The second most important genetic alteration is a co-deletion at chromosome regions 1p and 19q, which results from a chromosomal translocation t(1p;19q)(q10;p10), and is exclusively associated with ODGs and better prognosis [20]. Such examples demonstrate that the identification of new molecular genetic changes, which occur during glioma formation or development, are necessary to better guide clinical decision making.

Recently, mutations in the promoter region of the telomerase reverse transcriptase (*TERTp*) gene have been found in various cancers, including glioblastomas (GBMs) [18] and ODGs [3, 8]. Telomerase activity has a major role in tumorigenesis [6] and contributes to tumor development in different brain tumors, including astrocytomas, GBMs, and ODGs [12, 17]. The two most common mutations in the *TERTp* are C228T and C250T, which are located −124 base pairs (bp) and −146 bp respectively, upstream of the *TERT* ATG start site (chr5p15.33: 1,295,228 C > T and 1,295,250 C > T, respectively) [15]. These mutations confer unrestricted growth properties to tumor cells by elongation of telomere length because of *TERT* activation, which indicates the importance of its role in the anti-senescence and immortal cancer development [2, 7].

It has been reported that the effects of *TERTp* mutations are inversely correlated with mutations found in the *IDH*1 gene, [22] which is a well-known molecular factor for a favorable prognosis [27]. Therefore, several studies have independently proposed that mutations in *TERT*p are associated with poor prognosis [15]. However, recently Pekmezci et al. studied *TERTp* and *ATRX* mutation status and their prognostic value in large cohorts from the UCSF and Mayo clinics and The Cancer Genome Atlas (TCGA), and found that the wildtype (WT) *TERTp* group was associated with good prognosis only in *IDH1*/*IDH2* WT (*IDH*-WT) astrocytomas (grade II and III), while in other groups such as *IDH*-mutant astrocytomas (grades II and III) and GBMs (grade IV) with or without *IDH*-mutation, *TERTp* mutation status was not a statistically significant prognostic factor [23]. In addition, they found that *ATRX* was a good prognostic factor only in *IDH*-WT GBMs (grade IV), but not in any other glioma group [23].

In this study, we examined *TERTp* mutation status in a cohort of patients with either diffuse astrocytomas (grade III or IV) or ODG (grade II or III) to identify the frequencies of these mutations in our cohort and to evaluate their potential prognostic value, risk stratification, and future target therapy.

## Materials and methods

**Fig. 1:**
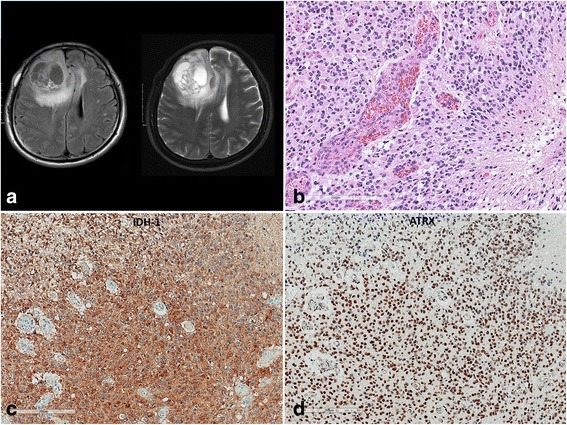
**a**) MRI showing a solid and cystic mass involving the right frontal lobe with diffuse increased T2 signal intensity in the right frontal lobe, insula, and corpus callosum genu with subfalcine herniation on the left side. **b**) The tumor is indicative of glioblastoma by the presence of necrosis and microvascular proliferation. **c**, **d**) Tumor cells are positive for IDH-1 (H09), R132H-mutant, and retained expression of ATRX

**Fig. 2:**
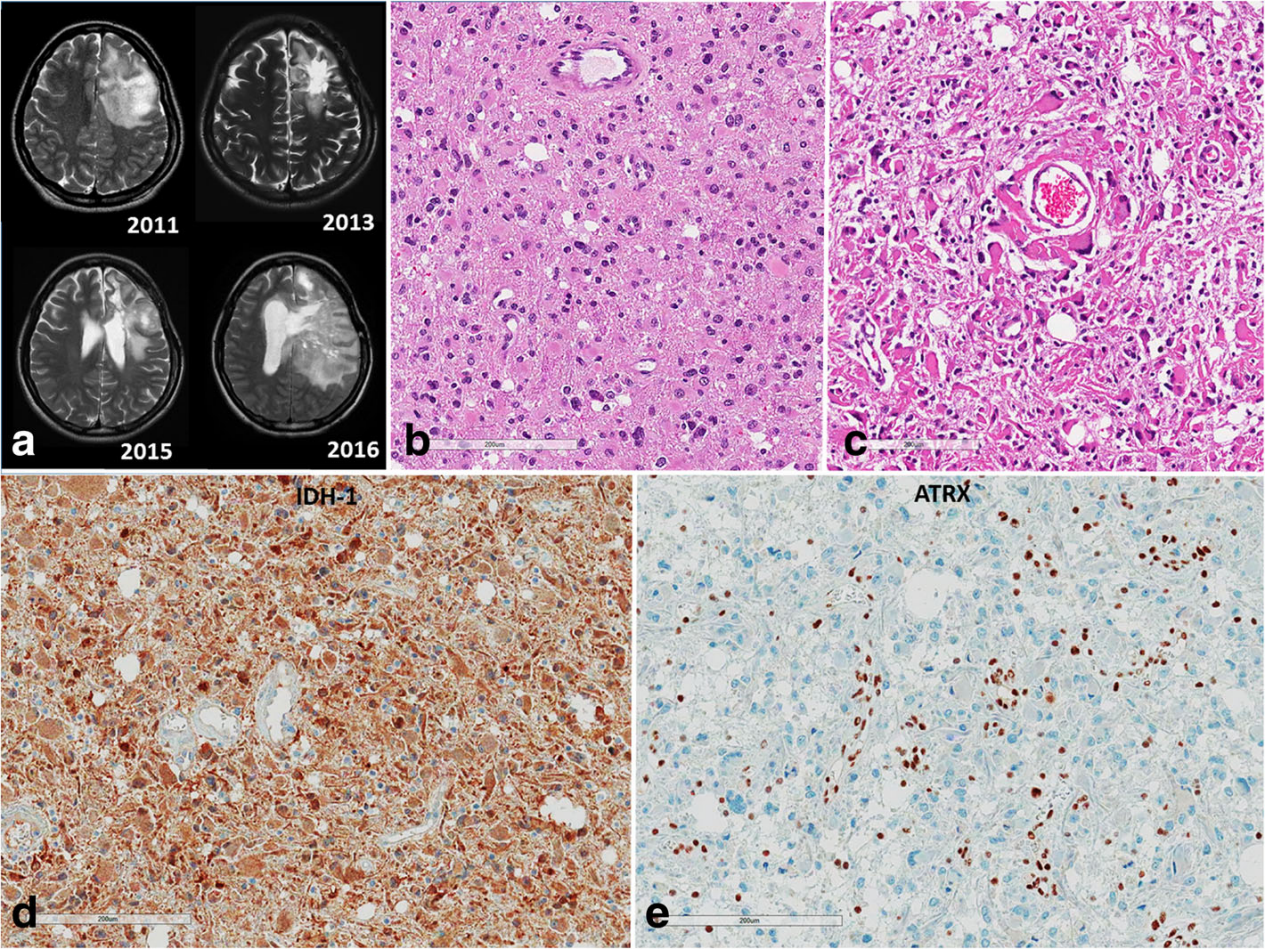
Recurrent anaplastic astrocytoma, *IDH*-mutant. **a**) MRI showing initial tumor and 3 times recurrent tumors despite concurrent chemotherapy and radiotherapy. **b**) Initial tumor showing a sheet of neoplastic gemistocytic astrocytes with mild to moderate nuclear atypia, WHO grade III. **c**) The second recurrent tumor shows more pleomorphic nuclei with bizarre tumor cell features than those of initial tumor, but there is neither tumor necrosis nor microvascular proliferation. **c**, **d**) However, note *IDH-1* and *ATRX* mutation were sustained (**c**: *IDH1 (H09)* staining, **d**: *ATRX* immunostaining)

Our cohort consisted of 168 patients in five groups, including 65 patients with ODGs carrying an *IDH*-mutation (*IDH*-mutant) and 1p/19q–codeletion (termed Group 1 or the ODG group), 23 patients with anaplastic astrocytoma (AA), *IDH*-mutation (Group 2), 13 patients with GBM, *IDH*-mutant (Group 3), 15 patients with AA, *IDH*-WT (Group 4), and 52 patients with GBM, *IDH*-WT (Group 5), which were obtained from the archives of the Department of Pathology of the Seoul National University Hospital (SNUH) from 2007 to 2015. All patients underwent surgery at the Department of Neurosurgery at the SNUH. All tumors were reviewed by two neuropathologists (SH Park and JK Won) according to the revised 2016 World Health Organization (WHO) classification of tumours of the CNS [19]. The mean age of patients with ODG, *IDH*-mutant AA, *IDH*-mutant GBM, *IDH*-WT AA, and *IDH*-WT GBM was 43.4, 42.6, 39, 48.6, and 55.3 years, respectively. The male-to-female ratio of these groups were 1:1, 7.4:2.6, 7:3, 4:6, and 6.6:3.4, respectively. Therefore, the ODG group had no gender predominance, but other subtypes had male predominance except for *IDH*-WT AA. Clinicopathological data were obtained from medical and pathological records, which are summarized in Table 1. Representative MRI, hematoxylin and eosin (H&E) features and immunohistochemical staining of IDH1 (H09) and ATRX are demonstrated in Figs. 1 and 2. This study followed the principles of the World Medical Association Declaration of Helsinki and it was approved by the Institutional Review Board of SNUH (IRB No.: 1307-093-505).

**Table 1:** Patients characteristics in our study cohort

Variable		Group 1 Oligodendroglioma, *IDH*-mutant and 1p19q–codeletion WHO grade II & III	Group 2 Anaplastic astrocytoma, *IDH*-mutant, WHO grade III	Group 3 Glioblastoma, *IDH*-mutant, WHO grade IV	Group 4 Anaplastic astrocytoma, *IDH*-wildtype, WHO grade III	Group 5 Glioblastoma *IDH*-wildtype WHO grade IV
	No	*n* (%)	*n* (%)	*n* (%)	*n* (%)	*n* (%)
Number of Patients	168	65 (100)	23 (100)	13 (100%)	15 (100%)	52 (100%)
Age (years)	Mean (range)	43.4 (21–65)	42.6 (21–65)	39 (29–49)	48.6 (26–72)	55.3 (27–79)
Gender	Male	33 (50.8)	17 (73.9)	9 (69.2)	6 (40)	35 (66.0)
	Female	32 (49.2)	6 (26.1)	4 (30.8)	9 (60)	17 (34.0)
Mean OS (mo)		38.0 (0–95)	59.4 (0.5–114.8)	40.0 (11.8–72.3)	28.2 (0.5–76.9)	19.6 (0.5–116)
Mean PFS (mo)		38.0 (1–96)	48.7 (0.5–114.8)	36.0 (9.5–68.7)	13.8 (0.5–76.9)	15.4 (0–103)
*TERT* sequencing	Wildtype	2 (3.1)	22 (95.6)	3 (23.1)	12 (80)	11 (21.2)
	Mutant	63 (96.9)	1 (4.4)	10 (76.9)	3 (20)	41 78.8)
1p, 19q FISH	No co-deletion	0 (0)	0 (0)	13 (100)	15 (100)	53 (100)
	Co-deletion	65 (100)	23 (100)	0 (0)	0 (0)	0 (0)
ATRX IHC	Wildtype (retained) Mutant (loss)	0 (0) 65 (100)	7 (30.4) 16 (69.6)	4 (30.7) 9 (69.2)	9 (60) 6 (40)	48 (92.3) 4 (7.7)
*MGMT* MSP	No methylation	11 (16.9)	9 (39.1)	3 (23.1)	7 (46.7)	26 (50.0)
	Methylation	54 (83.1)	14 (60.9)	10 (76.9)	8 (53.3)	26 (50.0)
Ki-67 labeling index (%)	Mean (range)	10.6 (1.5–39.5)	Not available	14 (1–39.3)	Not available	20.6 (0–54.8)

*Abbreviations*: *mo* month, *OS* overall survival, *PFS* progression free survival, *FISH* fluorescent in situ hybridization, *IHC* immunohistochemistry, *MSP* methylation-specific PCR, *HD* homozygous deletion, *WHO* World Health Organization

### Immunohistochemistry

Formalin-fixed paraffin-embedded (FFPE) tissue blocks were cut into 3-μm-thick slices and underwent immunohistochemistry (IHC). The DO-7 monoclonal antibody (mAb) for p53 (1:1000, DAKO, Glostrup, Denmark), sc48817 for Olig2 (1:500, Santa Cruz Biotechnology, Santa Cruz, US), the anti-human Ki-67 mAb (1:1000, clone MIB-1, DAKO), ATRX (Merck (Sigma-Aldrich), St Louis, US), and the mAb against the R132H mutation in *IDH*1 (1:100, clone H09, Dianova, Heidelberg, Germany) were used for accurate pathological diagnosis (Table 2). The appropriate positive controls were used and primary antibodies were omitted as negative controls.

**Table 2:** Primary antibodies used in this study

Name	Manufacturer	Clone	Dilution
Glial fibrillary acid protein	DAKO, Glostrup, Denmark	6F2	1:300
Olig2	Santa Cruz Biotechnology, Santa Cruz, US	sc48817	1:500
P53	DAKO, Glostrup, Denmark	DO7	1:1000
Ki-67	DAKO, Glostrup, Denmark	MIB-1	1:1000
IDH1	DIANOVA, Hamburg, Germany	DIAH09	1:100
ATRX	MERCK (Sigma), St Louis, USA	HPA001906	1:100

For antigen retrieval, cell conditioning with CC1 buffer pH 8.4 (Ventana, Export, PA) was used for all antibodies

Virtual microscope scanning was performed on Ki-67-, p53-, and Olig2-immunostained slides using the Aperio Spectrum Plus image analyzer (Leica Biosystems Nussloch GmbH, Nußloch, Germany) and we used them as an aid when reviewing the diagnosis. Intact tumor areas of each slide, excluding areas with crush artifacts, necrosis, and other poor-quality regions, were evaluated. For automated quantitation of each marker, nuclear V9 IHC algorithms of the Aperio Spectrum Plus image analyzer were applied after validating the data from comparisons to manual counts in a number of representative cases. The proportion of cells expressing Ki-67, p53, and Olig2 in the nuclei was calculated as a percentage of the total number of tumor cell-nuclei. We used a 30% cut off for p53 positivity.

### Fluorescence in situ hybridization for 1p and 19q

FFPE blocks underwent fluorescence in situ hybridization (FISH). Paired fluorescein isothiocyanate (FITC)/rhodamine-labeled DNA probes for chromosome regions 1p (LSI1p36/LSI1q25) and 19q (LSI19q13/LSI19p13) were used for deletion analysis.

Green and red fluorescent signals were counted under a BX01 fluorescence microscope (Olympus, Olympus corporation, Japan) using the MetaMorph® Imaging System (Universal Imaging, Molecular Devices, US). For each hybridization, the number of green and red signals was assessed using a minimum of 100 non-overlapping nuclei. An interpretation of a deletion was made when >50% of nuclei harbored a single red or green signal. This determination was based on the frequency of non-neoplastic nuclei that contained one signal (median ± 3 standard deviations) using the same probes in non-neoplastic control (seizure-resection) specimens.

### *IDH1*/*IDH2* and *TERT* mutation analysis

Sanger sequencing was used to determine the frequency of mutations in *IDH1*, *IDH2*, and *TERT*. Primer design was based on sequence data from accession numbers NM 005896 for *IDH1* and NM 002168 for *IDH2* (http://www.ncbi.nlm.nih.gov). Representative H&E stained slides were reviewed by two pathologists (SH Park and JK Won), and a selected tumor region was macrodissected manually from consecutive FFPE sections. After deparaffinization, genomic DNA was extracted and underwent nested-PCR for *IDH1* (186 bp), *IDH2* (302 bp), and the *TERT*p. PCR primer sequences used for *IDH1* and *IDH2* are summarized in Table 2. PCR amplification and Sanger sequencing was performed using an ABI-PRISM 3730 DNA Analyzer (Applied Biosystems, Vernon Hills, IL, USA). PCR was performed in 40 μL reaction conditions that included standard buffer conditions, 200 ng of DNA, and GoTaq DNA Polymerase (Promega, Madison, WI, USA). PCR amplification for *IDH1* consisted of 45 cycles with denaturation at 95 °C for 30 s, followed by annealing at 62 °C for 30 s, and extension at 72 °C for 1 min, whereas PCR amplification for *IDH2* consisted of 40 cycles with denaturing at 95 °C for 30 s, followed by annealing at 55 °C for 30 s, and extension at 72 °C for 1 min. Two microliters of PCR amplification product were sequenced using the BigDye Terminator v3.1 Cycle Sequencing Kit (Applied Biosystems, Foster City, CA, USA). Twenty-five cycles were performed using 12 ng of the sense primers IDH1f 5′-M13-GTAAAACGACGGCCAGTCGGTCTTCAGAGAAGCCA-3′ or IDH2f 5′-GCTGCAGTGGGACCACTATT-3′ with denaturation at 96 °C for 10 s, annealing at 50 °C for 5 s, and extension at 60 °C for 4 min.

Two hotspot mutations, C228T and C250T, in the *TERT*p were screened using the oligonucleotide primers shown in Table 3. M13 has a universal sequencing primer site with the sequence 5′-GTAAAACGACGGCCAGT-3′ that was used for PCR amplification of the proximal *TERTp* for Sanger sequencing using standard methods. PCR was performed in 50 μL reaction mixtures containing 5 μL of DNA, 10 mM of each dNTP, 10 pmole/μL each primer, 5X Band Doctor™, 10X h-Taq Reaction buffer (15 mM MgCl_2_ mixed), and 2.5 U/μL of Solg™ h-Taq DNA Polymerase. PCR was initiated at 95 °C for 15 min, followed by 45 cycles of 95 °C for 30 s, 62 °C for 30 s, and 72 °C for 1 min with a final extension of 72 °C for 7 min.

**Table 3:** Primer sequences of *IDH1/IDH2, TERT* promoter, and *MGMT* for Sanger sequencing

	Prime		
Gene	Forward	Reverse	Product (bp)
*IDH1*	5′-M13-GTA AAA CGA CGG CCA GTC GGT CTT CAG AGA AGC CA-3′	5′-GCG GAT AAC AAT TTC ACA CAG GGC AAA ATC ACA TTA TTG C-3′	180–190
*IDH2*	5′-GCT GCA GTG GGA CCA CTA TT-3′	5′-TGT GGC GTT GTA CTG CAG AG-3′	295–305
*TERT*	5′-M13-GTA AAA CGA CGG CCA GTC ACC CGT CCT GCC CCT TCA CCT T-3′ (M13: 5′-GTA AAA CGA CGG CCA GT-3′)	5′-GCA CCT CGC GGT AGT GG-3′	300–310
*MGMT*	5′-TTT CGA CGT TCG TAG GTT TTC GC-3′	5′-GCA CTC TTC CGA AAA CGA AAC G-3′	80–90

### Methylation-specific PCR

Methylation status of the O-6-methylguanine-DNA methyltransferase (*MGMT*) gene promoter was determined by extracting tumor genomic DNA from FFPE sections for bisulfite conversion using the EZ DNA methylation-Gold Kit (Zymo Research, Orange County, CA, USA). *MGMT* methylation-specific PCR was performed using primer pairs specific to methylated and unmethylated *MGMT* promoter sequences (Table 3).

### Statistical analysis

All statistical analyses were performed using SPSS software v23 (IBM Corp., New York, NY, USA). Comparisons between variables were performed using the χ^2^ test, Fisher’s exact test, or the Student’s t-test. Disease-free survival (DFS) was measured from the date of surgery to that of disease recurrence or onset of metastasis. OS was measured from the date of diagnosis until death from any cause. Survival analysis was performed using the Kaplan-Meier method with log-rank test. Multivariate Cox regression analysis was performed with consideration of co-linearity. Two-sided *P*-values <0.05 were considered statistically significant.

## Results

### *TERTp* mutation status and relationship with clinicopathological parameters

**Fig. 3:**
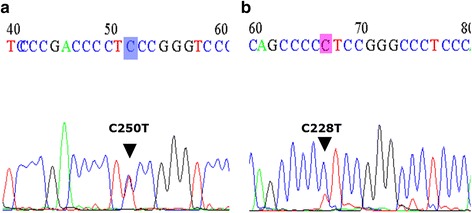
Electropherograms showing sequence of the *TERT* promoter region with the two hot-spot mutations **a**) C250T and **b**) C228T

Somatic *TERTp* mutations were analyzed by direct Sanger sequencing. *TERTp* mutations were detected in 96.9%, 4.4%, 76.9%, 20.0% and 84.6% of patients in Group 1, 2, 3, 4, and 5, respectively. The C228T mutation was much more frequent than the C250T mutation in our cohort (Fig. 3 and Table 4). There was no evidence of an association between *TERTp* mutation and *IDH* or *ATRX* mutation (ATRX loss) or *MGMTp* methylation status, gender preference, or age preference in all patient groups except the patients with AA (grade III) by Pearson χ^2^ test. Further, in patients with AA (combined groups 2 and 4), the *TERTp* mutation was more common for over 55 years of age (*P* = 0.004), which may be the reason why *TERTp* mutation is an adverse marker specifically for *IDH*-WT AA. In this study we could not reach statistical significance (*P* = 0.113), because of the lack of the number of *TERTp* mutant cases (Fig. 5c).

**Table 4:** The frequency of *TERTp* mutations

Diagnosis	C228T mutation	C250T mutation	Both *TERT* mutations
	No. (%)	No. (%)	No. (%)
Oligodendroglioma	55/63 (87.3)	8/63 (12.7)	63/65 (96.9)
AA, *IDH*-mutant	1/1 (100)	0/1 (0)	1/23 (4.4)
GBM, *IDH*-mutant	7/10 (70)	3/10 (30)	10/13 (76.9)
AA, *IDH*-wildtype	2/3 (66.7)	1/3 (33.3)	3/15 (20.0)
GBM, *IDH*-wildtype	36/44 (81.8)	8/44 (18.2)	44/52 (84.6)

*Abbreviations*: *AA* anaplastic astrocytoma, *GBM* glioblastoma, *TERTp*, *TERT* promoter region

### *IDH1/IDH2* mutation status and relationship with clinicopathological parameters

The R132H mutation in *IDH1* was found in 60.5% (23/38) of patients with AA and 20.0% (13/65) of our patients with GBM (Groups 3 and 5), whereas all patients with ODG (Group 1) had a mutation either in *IDH1* (*n* = 62) or *IDH2* (*n* = 3), as determined by Sanger sequencing. Correlation analysis between *IDH* mutation and clinicopathological features in these five groups of gliomas revealed that *IDH* mutation was associated with a younger age at diagnosis and in patients with *MGMT* methylation and *ATRX* mutation, respectively by Pearson χ^2^ test, but it was not correlated with *TERTp* mutation (*p* < 0.05). 

### *MGMTp* methylation and *ATRX* mutation (ATRX loss) status

*MGMTp* methylation was present in 83.1% 60.9%, 76.9%, 53.3%, and 49.1% of cases from Groups 1, 2, 3, 4, to 5, respectively. An *ATRX* mutation was present in 69.6% 69.2%, 40.0%, and 5.7% of patients from Group 2, 3, 4, to 5 respectively, but it was not found in patients with ODGs (Group 1). *MGMTp* methylation was more common in *IDH*-mutant GBMs, but was not associated with *IDH* mutation status in AA. ATRX mutation was also more common in IDH-mutant GBMs and/or younger patient under 55 years old with GBM.

### Prognostic impact of *TERTp, ATRX, and IDH* mutations, and *MGMTp* methylation

**Fig. 4:**
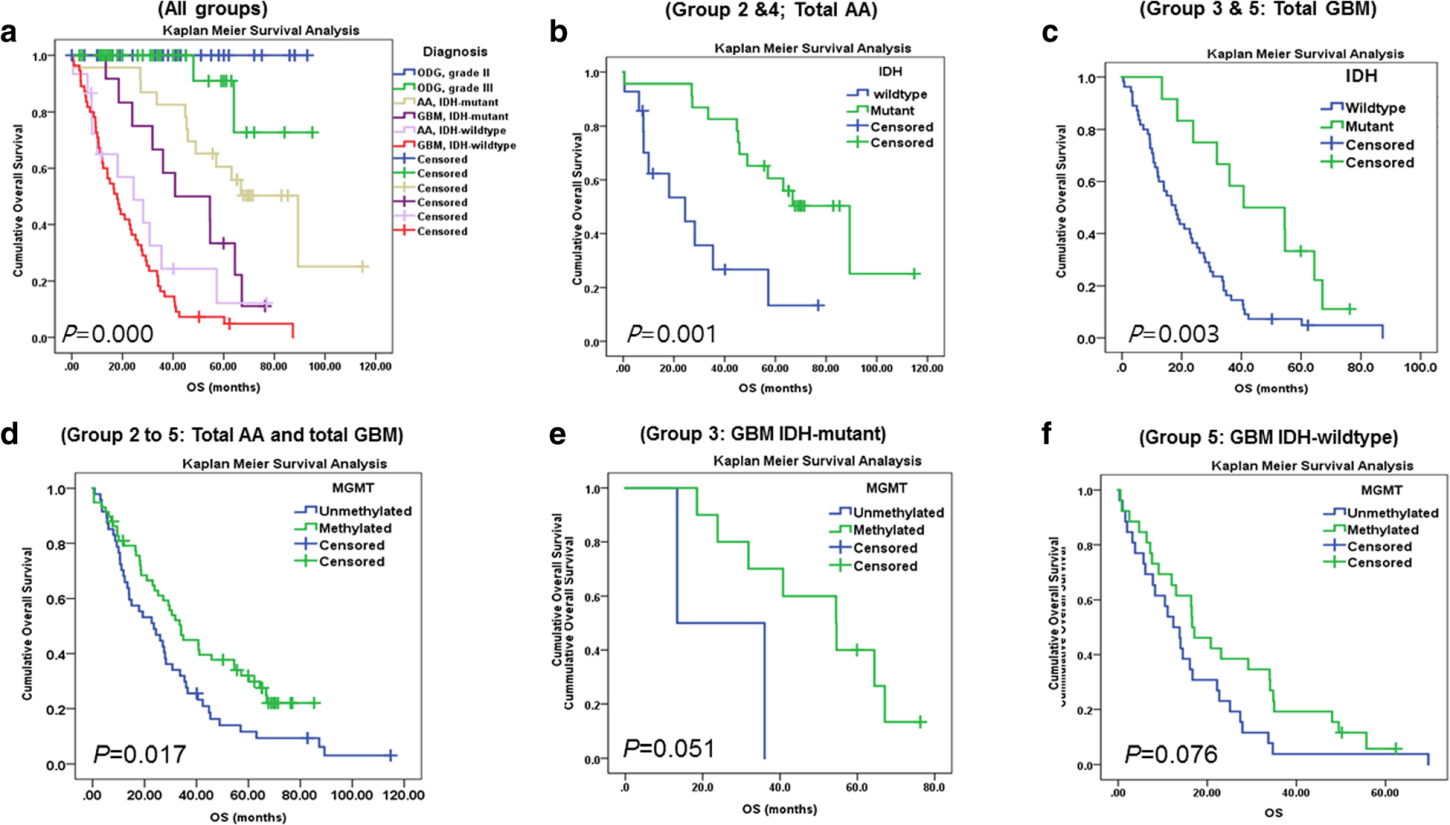
**a**) All gliomas included in this study showed statistically different survival. **b**) Among patients with grade III anaplastic astrocytoma (AA), the *IDH*-mutant group showed remarkably better survival than *IDH*-WT group. **c**) The *IDH*-mutant grade IV GBM group had significantly better survival compared to the *IDH*-WT GBM group. **d**) Of all patients with grade III and IV astrocytic tumors, *MGMTp-*methylated groups have statistically better survival (*P* = 0.017). **e** and **f**) However, when GBM is subdivided into *IDH*-mutant and *IDH*-WT, *MGMTp-*methylation has a modest survival effect (*P* = 0.051 and *P* = 0.076, respectively). These results indicate there is no bias effect associated with our group

Using Kaplan-Meier survival analysis, we found that these five groups were well segregated (*P* = 0.000) (Fig. 4a) and patients with *IDH*-mutant gliomas had significantly better survival compared to those with *IDH*-WT gliomas (*P* < 0.001, *P* < 0.003) (Fig. 4b and c).

In addition, we found that *MGMTp* methylation was a good prognostic factor in pooled groups with total GBM (Groups 3 and 5) (*P* = 0.008) and total AA and GBM groups (groups 2, 3, 4, and 5 combined) (*P* = 0.017) (Fig. 4d); however, in individual groups of gliomas, *MGMTp* methylation was not correlated with OS. In *IDH*-mutant and *IDH*-WT GBMs (Groups 3 and 5), we found that *MGMTp* methylation was a borderline indicator of better prognosis (*P* = 0.051 and 0.076) (Fig. 4e-f). In total AA (Groups 2 and 4), *MGMTp* methylation was not correlated with survival (*P* = 0.164).

**Fig. 5:**
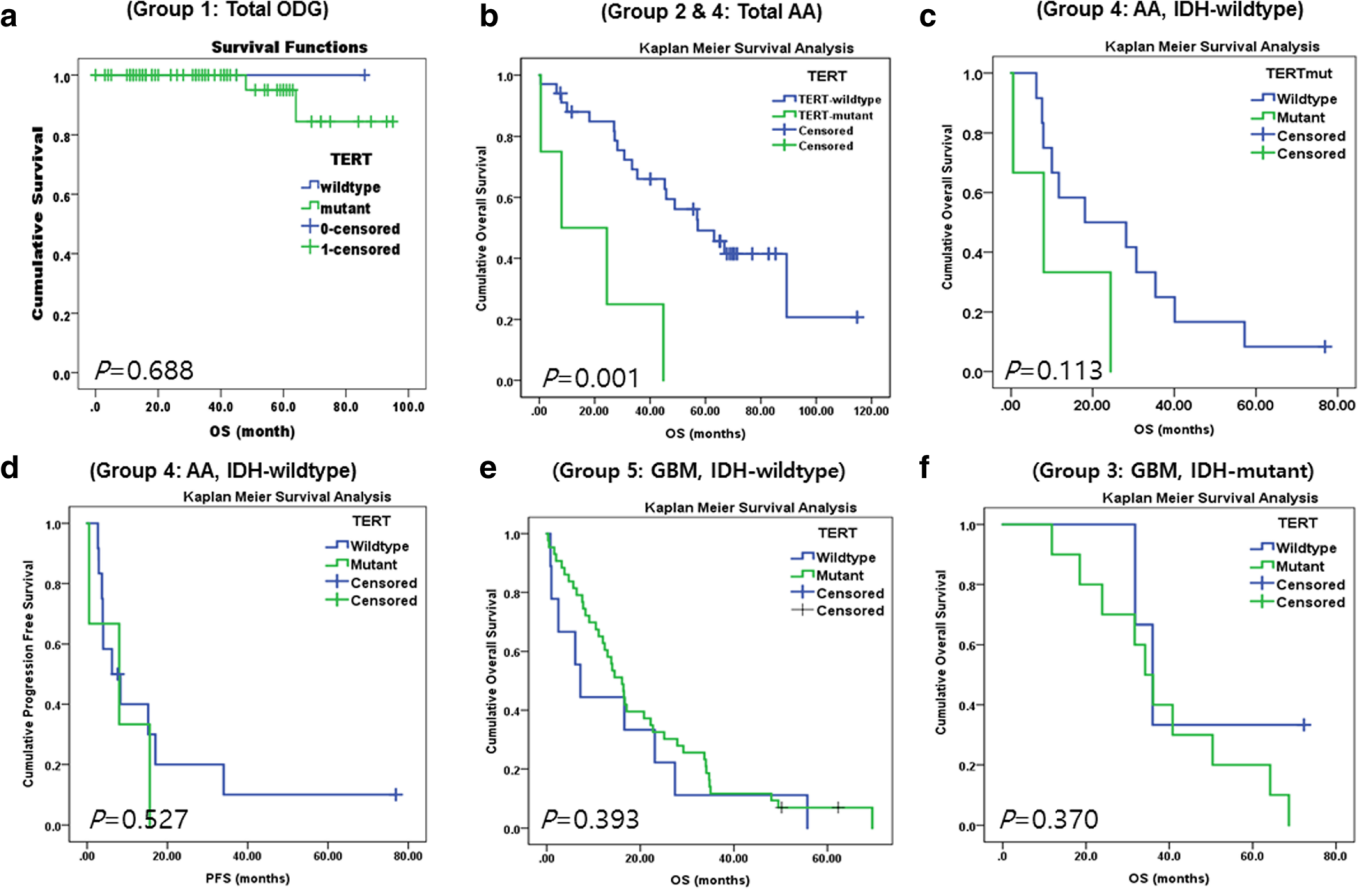
**a**) In group 1 (ODG), we found that *TERTp* mutations were not associated with OS (*P* = 0.688). **b**) In patients with combined group 2 and 4 (total AAs), *TERTp-* mutant had poorer survival (*p* = 0.001) than *TERT*p-WT patients, due to *TERTp* mutation was more common in poor prognostic *IDH-*WT AA and older age over 55 years old. **c**) In patients with grade III *IDH*-WT AA, the *TERTp*-mutant group shows poorer OS compared to the *TERTp*-WT group but was not statistically significant due to shortage of the number of *TERTp* mutant case (*p* = 0.113), **d**) and has no effect on PFS (*p* = 0.527). **e**) In the Kaplan-Meier survival analysis, the *TERTp*-mutation status in patients with grade 4 *IDH*-WT has no effect on the patient’s OS (*P* = 0.393). **f**) A similar finding is seen in the *IDH*-mutant GBM groups (*P* = 0.370)

In group 1 (ODG), we found that *TERTp* mutations were not associated with either OS or PFS (*P* = 0.688 and *P* = 0.427, respectively) (Fig. 5a). In a combined cohort with Groups 2 and 4, *TERTp* mutation was strongly associated with patient survival (Fig. 5b), although this finding may be attributed to the *TERTp* mutation being more frequent in the *IDH*-WT AA group, which has the worse prognosis than *IDH*-mutant AA.

**Fig. 6:**
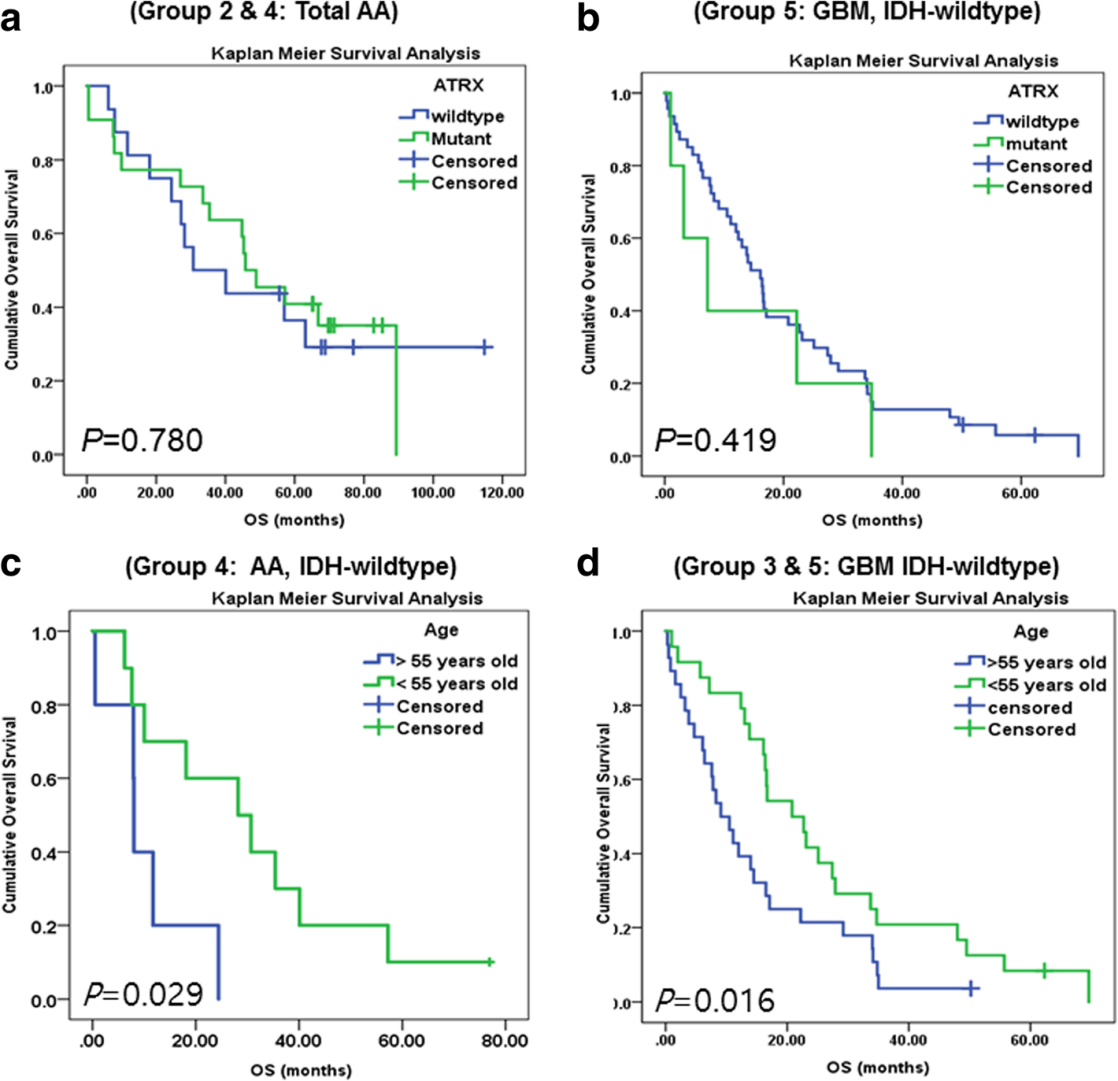
*ATRX* mutation was not a statistically significant biomarker in all grade III and IV astrocytic tumors. **a**) In cases with grade III anaplastic astrocytoma, combined *IDH*-mutant and –WT tumors, the *ATRX*-mutant group does not show statistically different OS compared to the *ATRX*-WT group (*P* = 0.780), **b**) a finding which is comparable to that found in the GBM *IDH*-WT group (*P* = 0.419). **c**, **d**) Statistical significance of age were obtained in the group 4 (AA, IDH-wildtype) (*P* = 0.029), and the group 5 (*IDH*-WT GBMs) (*P = 0.016*), patients older than 55 years have a statistically significant worse OS compared to patients younger than 55 years of age

In Group 4 *(IDH*-WT AA), we found that patients with *TERTp-*mutant tumor showed shorter median survival compared to patients with *TERTp*-WT tumors [median survival with 95% CI: 8.0 months (0.000–20.002) vs. 18.1 months (0.000–46.107), respectively]; however, *TERTp* mutation status did not have an statistically significant effect on OS or PFS (Table 5, Fig. 5c and d), possibly because the number of *TERTp* mutant cases were too few to attain statistical significance (*P* = 0.113). When we evaluated the prognostic value of *TERTp* mutations in each of the five groups by Cox regression analysis, we found no evidence of association, with the exception of Group 5 (*IDH*-WT GBM), in which we found by multivariate analysis that patients with *TERTp* mutation had statistically worse prognosis than patients with *TERTp-*WT (*P* = 0.045) [median survival with 95% CI: 16.4 months (13.012–19.516) vs. 13.0 months (1.021–25.039), respectively] (Table 5). In addition, we found that having an *ATRX* mutant tumor did not affect patient OS or PFS in any of the five groups (all *P* > 0.05) (Fig. 6a and b).

**Table 5:** The result of multivariate analysis on group 4 and 5

4	*N* (%)	Age median (range)	Median survival years (95% CI)	Univariate analysis			Multivariate analysis		
				Hazard ratio	95% CI	*p* Value	Hazard ratio	95% CI	*p* Value
Group 4									
AA, *IDH*-wildtype	15	51 (26–72)	18.1 (0.000–36.278)						
*TERTp*-mutant	3	69.0 (62–72)	8.0 (0.000–20.002)	0.341	0.85–1.377	1.113	1.11	0.116–10.608	0.928
*TERTp*-wildtype	12	46.5 (27–58)	18.1 (0.000–46.107)						
ATRX-mutant	9	44.5 (27–69)	28.2 (0.954–55.446)	0.555	0.182–1.687	0.299	0.731	0.214–2.498	0.618
ATRX-wildtype	6	54 (41–72)	11.7 (6.733–16.667)						
*MGMTp*-methylated	8	48.5926–62)	28.2 (18.448–37.952)	1.109	0.365–3.367	0.856	1.076	0.261–4.434	0.919
*MGMTp*-unmythylated	7	54 (27–72)	7.9 (2.218–13.582)						
Age < 55 years old	10	41.5 (26–54.9)	28.2 (8.676–47.724)	4.027	1.048–15.472	0.043	0.296	0.042–2.107	0.224
Age > 55 years old	5	62 (57–69)	8.0 (7.785–8.215)						
Group 5									
GBM, *IDH*-wildtype	52	55 (17–79)	14.5 (10.378–18.622)						
*​TERTp*-mutant	41	58 (36–79)	16.4 (13.012–19.516)	0.768	0.390–1.513	0.445	2.111	1.016–4.389	0.045
*​TERTp*-wildtype	11	42 (31–69)	13.0 (1.021–25.039)						
ATRX-mutant	5	58 (29–69)	13.0 (0.513–25.547)	0.682	0.269–1.733	0.422	1.269	0.499–3.231	0.617
ATRX-wildtype	47	55 (31–79)	16.4 (12.773–20.027)						
*MGMTp*-methylated	26	57.5 (29–79)	18.3 (12.671–23.989)	0.579	0.326–1.031	0.061	0.665	1.399–5.078	0.002
*MGMTp*-unmythylated	26	51 (36–79)	13.0 (9.407–16.653)						
Age < 55 years old	24	45 (29–54.9)	20.8 (13.599–28.001)	1.843	1.047–3.331	0.035	3.090	1.578–6.049	0.001
Age > 55 years old	28	67 (55.3–79)	10.5 (5.703–15.297)

From Kaplan Meier survival analysis of diffuse astrocytic tumors of grade III and IV, we found that *IDH*-mutant and younger age-patients (under 55 years of age) (Figs. 4b-c and 6d) were associated with statistically significant better survival compared to their counter parts. In the *IDH*-mutant groups (Group 2 and Group 3), because most patients were younger than 55 years of age, age was not a factor for patient survival (Table 1) and particularly in GBMs (combined group 3 and group 5), the *IDH*-mutation was exclusively present in the patients younger than 55 years old.

On multivariate Cox regression analysis of the Group 5 (*IDH*–WT GBMs), *TERT*-mutant (*P* = 0.045), *MGMT*p methylation (*P* = 0.003) and younger age under 55 years old (*P* = 0.001) were statistically significant good prognostic factors (Table 5), but in other groups, patients with *TERTp* mutation, *ATRX* mutation, *MEMTp* methylation and younger age did not show significantly different survival rate than counter parts. Regarding age-related factors, no evidence of statistical significance was found because of the small number of patients aged 55 years or older with Type III astrocytomas (Fig. 6c).

## Discussion

Telomerase is a specialized reverse transcriptase that maintains telomere length [10]. Telomerase activity is robustly expressed in embryonic cells, while it is suppressed in fully matured somatic cells during adult life. However, it is expressed in approximately 85% of solid tumors and most immortalized cell lines. Recently, several studies have reported that *TERTp* mutations are frequently found in gliomas, especially in ODGs and GBMs, which results in altered telomere lengthening and lead to prolonged longevity of tumor cells by escaping from the tumor cell senescence [3]. Aita et al. [19] found that *TERTp* mutations are present in >70% of patients with ODG and GBM, and that the frequency of *TERTp* mutation is even higher than previous reports in ODG and GBM, because the diagnostic criteria of diffuse glioma became more strict with the integration of genetics in the diagnosis according to the revised 2016 WHO classification criteria.

Based on this finding, we studied the frequency and putative prognostic importance of mutations in *TERTp* and *ATRX* as well as *MGMTp* methylation in five patient groups, which were classified by 2016 revised WHO classification of CNS tumors: Group 1: ODGs, Group 2: grade III AA (*IDH*-mutant), Group 3: grade IV GBM (*IDH*-mutant), Group 4: grade III AA (*IDH*-WT), and Group 5: grade IV GBM (IDH-WT) [19]. These five groups were well-classified on the basis of OS rate by Kaplan Meier Survival analysis (Fig. 4a). Patients with *IDH*-mutant GBM showed better survival compared to those with *IDH*-WT AA and *IDH*-WT GBM; however, they showed worse OS than patients with *IDH*-mutant AA. The OS of patients with *IDH*-WT AA was comparable to that found in patients with *IDH*-WT GBM. These findings verified our cohort was not deviated groups.

Furthermore, we found that the *TERTp* mutation frequencies in these five groups were 96.9%, 4.4%, 76.9%, 20%, and 84.6%, respectively (Table 4). Therefore, patients with grade III *IDH*-mutant AA (Group 2) had the lowest incidence of *TERTp* mutation (4.4%) and patients with *IDH*-WT grade III AA (Group 4) had the second lowest frequency of *TERT*p mutation (20%). These frequencies about half of those reported by Eckel-Passow et al. [9]. Among their series of grade II or III gliomas (*N* = 586), 40.4% (40/99) of IDH-WT astrocytoma and 10.1% (31/306) of IDH-mutant astrocytoma was *TERT*p-mutated tumors and remained 181 cases were triple positive (1p/19q co-deleted, *IDH*-mutant, and *TERT*-mutated) ODG [9]. TERT mutation only cases (that means *IDH*-WT astrocytomas) were the worst prognostic group and that group had worse survival than that of patient with *IDH*-WT and *TERT*-WT [9].

In addition, we found increased frequencies of *TERT*p mutations in patients with ODGs and GBMs, both *IDH-*mutant and *IDH-*WT, which is comparable to those found in a previous report [14]. Therefore, similar to previously reported studies, we propose that telomerase activation may be an underlying mechanism in these gliomas, especially in cases of ODG and GBMs [5]. In addition, we found that occurrence of the C228T and C250T mutations were mutually exclusive in our cohort, and that C228T was more common than C250T (Table 4) [21].

In previous studies, the 1p/19q co-deletion was strongly associated with mutations in *TERTp* [13, 25]. In the present study, we found that 96.9% of patients with ODG had a *TERTp* mutation, whereas two patients with ODG were *TERTp-*WT (Table 4). However, in cases with GBM that did not harbor the 1p/19q co-deletion, we found that they had a high frequency of *TERTp* mutation, and therefore, conclude that the *TERTp* mutation is not exclusively associated with the 1p/19q co-deletion.

Regarding *TERTp* mutation from a prognostic perspective in diffuse gliomas, previous studies showed conflicting results. Labussiere et al. found that *TERTp* mutations may be associated with poorer outcome in high-grade gliomas, [16] however, Pekmezci et al. reported that *TERT*-mutants had significantly worse survival only in *IDH*-WT astrocytoma, which includes grades II and III [23]. Such contradictory effects of *TERTp* mutation on patient outcome between groups have been reported previously [9]. Abudumijiti et al. found comparable results to those of Pekmezci et al. and concluded that adult *IDH*-WT lower-grade gliomas should be further classified by *TERTp* mutation status [1]. Similarly, our detailed analysis using group classifications according to *IDH*-mutation status and multivariate analysis revealed that only in those cases with grade IV *IDH*-WT GBM, *TERTp*-mutation was associated with worse OS compared to *TERTp*-WT (Table 5). We did not study grade II diffuse astrocytoma because we wanted a homogeneous group of grade III cases and to not be potentially biased by grading. In *IDH*-mutant AA, the number of *TERTp*-mutant cases was too small to evaluate its effect, while in grade IV GBMs, regardless of *IDH*-mutation status, *TERTp*-mutation did not affect patient OS (Fig. 5c and d). However, it was difficult to verify this finding in patients with ODG because of the high frequency of *TERTp* mutations (96.9%) in this tumor group. In addition, we found that the *MGMTp* methylation group had better survival in combined grade III and IV astrocytic tumors and in both *IDH*-mutant and *IDH*-WT GBMs; however, we found that *ATRX* mutation did not confer a prognostic effect in any of the five groups evaluated in our study, which is different from Pekmezci et al.’s conclusion [23].

Our findings differ from those of Eckel-Passow et al. [9]. They concluded that the patients with grade II or III gliomas with *TERTp* mutation only revealed the worst survival, but *TERTp* mutation status was not correlated with survival rate of patients with grade IV glioma. This result means *TERTp* mutation may be poor prognostic marker only in *IDH*-WT grade II or III astrocytomas.

Further studies incorporating such classification using molecular alterations including *TERTp* mutations are warranted to determine their putative prognostic or diagnostic importance for gliomas.

Because cancer cells acquire immortality by telomere lengthening, the telomerase enzyme is a likely anticancer therapeutic target [10, 24]. Daniel et al. [24] proposed that the human immunodeficiency virus reverse transcriptase inhibitor azidothymidine (AZT) acts as a telomerase inhibitor. In addition, several telomerase-targeting inhibitors such as the antisense oligonucleotide inhibitor GRN163L and immunotherapies that use dendritic cells (GRVAC1), the hTERT peptide (GV1001), or cryptic peptides (Vx-001) are under investigation [24].

## Conclusion

In conclusion, mutations in the promoter of *TERTp* are common in patients with GBM or ODG. In terms of survival effect, mutations in *IDH1* and *MGMTp* methylation and age under 55 were associated with favorable prognosis in diffuse gliomas and in cases with grade III AA (combined *IDH*-mutant and *IDH*-WT AA), *TERTp* mutant carriers had worse OS than *TERTp* WT carriers. In multivariate analysis, *TERTp* mutation was strongly correlated with poor outcome in patients with *IDH*-WT GBM, suggesting that it may be of prognostic value in this subgroup of patients with gliomas.

In ODGs, because all tumors have a *TERTp* mutation except for two casess, we could not evaluate the putative prognostic value using the mutation status of this gene.

## Abbreviations

AA: Anaplastic astrocytoma
ATRX: Alpha-thalassemia/mental retardation syndrome, X-linked
AZT: Azidothymidine
CNS: Central nervous system
GBM: Glioblastoma
*IDH*: Isocitrate dehydrogenase
*MGMTp*: O6 methylguanine-DNA methyltransferase promoter
MRI: Magnetic resonance imaging
ODG: Oligodendroglioma
OS: Overall survival
PFS: Progression free survival
*TERTp*: Telomerase reverse transcriptase promoter
WHO: World Health Organization
WT: Wildtype
